# Engineering and Dissecting the Glycosylation Pathway of a Streptococcal Serine-rich Repeat Adhesin*

**DOI:** 10.1074/jbc.M116.752998

**Published:** 2016-11-14

**Authors:** Fan Zhu, Hua Zhang, Tiandi Yang, Stuart M. Haslam, Anne Dell, Hui Wu

**Affiliations:** From the Departments of ‡ Pediatric Dentistry and; § Microbiology, University of Alabama at Birmingham, Schools of Dentistry and Medicine, Birmingham, Alabama 35244 and; the ¶ Department of Life Sciences, Imperial College London, London SW7 2AZ, United Kingdom

**Keywords:** bacterial adhesion, glycoprotein, glycoprotein biosynthesis, glycosylation, glycosyltransferase, Streptococcus parasanguinis, glycoengineering, rhamnosyltransferase, serine-rich repeat protein

## Abstract

Serine-rich repeat glycoproteins (SRRPs) are conserved in Gram-positive bacteria. They are crucial for modulating biofilm formation and bacterial-host interactions. Glycosylation of SRRPs plays a pivotal role in the process; thus understanding the glycosyltransferases involved is key to identifying new therapeutic drug targets. The glycosylation of Fap1, an SRRP of *Streptococcus parasanguinis*, is mediated by a gene cluster consisting of six genes: *gtf1*, *gtf2*, *gly*, *gtf3*, *dGT1*, and *galT2*. Mature Fap1 glycan possesses the sequence of Rha1–3Glc1-(Glc1–3GlcNAc1)-2,6-Glc1–6GlcNAc. Gtf12, Gtf3, and dGT1 are responsible for the first four steps of the Fap1 glycosylation, catalyzing the transfer of GlcNAc, Glc, Glc, and GlcNAc residues to the protein backbone sequentially. The role of GalT2 and Gly in the Fap1 glycosylation is unknown. In the present study, we synthesized the fully modified Fap1 glycan in *Escherichia coli* by incorporating all six genes from the cluster. This study represents the first reconstitution of an exogenous stepwise *O-*glycosylation synthetic pathway in *E. coli*. In addition, we have determined that GalT2 mediates the fifth step of the Fap1 glycosylation by adding a rhamnose residue, and Gly mediates the final glycosylation step by transferring glucosyl residues. Furthermore, inactivation of each glycosyltransferase gene resulted in differentially impaired biofilms of *S. parasanguinis*, demonstrating the importance of Fap1 glycosylation in the biofilm formation. The Fap1 glycosylation system offers an excellent model to engineer glycans using different permutations of glycosyltransferases and to investigate biosynthetic pathways of SRRPs because SRRP genetic loci are highly conserved.

## Introduction

Protein glycosylation is an important post-translational modification that mediates a variety of biological processes. It consists of the covalent linkage of glycans either to the amide nitrogen of Asn residues (*N*-glycosylation) or to the hydroxyl oxygen of Ser or Thr residues (*O*-glycosylation) (1). This modification occurs across all domains of life (2). Serine-rich repeat glycoproteins (SRRPs)^3^ are a family of glycosylated adhesins highly conserved in Gram-positive bacteria that mediate bacterial-host interactions (3). They have been implicated in the bacterial pathogenesis in a variety of infectious diseases such as pneumonia, infective endocarditis, meningitis, and oral infectious diseases (3). *Streptococcus parasanguinis*, one of the early colonizers in the oral cavity, plays an important role in sub-acute bacterial endocarditis (4). The adhesion of *S. parasanguinis* is mediated by its long, peritrichous fimbriae (5). Fap1, the first identified SRRP, is the major subunit of the long fimbriae (5) and crucial for the biofilm formation of *S. parasanguinis*. Fap1-like SRRPs (6), including GspB and Hsa from *Streptococcus gordonii*, PsrP of *Streptococcus pneumoniae*, Srr-1 and Srr-2 of *Streptococcus agalactiae*, SraP of *Streptococcus aureus*, and SrpA of *Streptococcus sanguis* (7–13), have also been implicated in bacterial fitness and virulence (7–13). Therefore, the understanding of how bacteria produce this family of proteins and how they contribute to bacterial pathogenesis is key to understanding bacterial-host interactions and identifying new targets for antimicrobial drug discovery.

The biogenesis of Fap1 is composed of two major steps: glycosylation and secretion. Glycosylation of Fap1 is critical for bacterial adhesion and biofilm formation (6, 14). The Fap1 glycosylation has been studied extensively and emerged as a model system to investigate stepwise *O-*linked protein glycosylation in bacteria (6, 15–20). We have determined that the mature Fap1 carries *O-*linked hexasaccharides with the sequence of rhamnose-1–3-glucose 1-(glucose 1–3 *N*-acetyl glucosamine 1)-2,6-glucose1–6 *N*-acetyl glucosamine (Rha1–3Glc1-(Glc1-3GlcNAc1)-2,6Glc1–6GlcNAc) (15).

Two genes located downstream of the *fap1* locus, *gtf1* and *gtf2*, encode a two-protein enzyme complex Gtf12, which initiates the first step of the Fap1 glycosylation by transferring GlcNAc to the serine residues of the Fap1 polypeptide backbone (21). Upstream of the *fap1* locus, there is a gene cluster including *gly*, *gtf3*, d*GT1*, and *galT2* (22). We have identified Gtf3 and dGT1 as *bona fide* glycosyltransferases (15, 17). Gtf3 is a classical glucosyltransferase (GlcT) transferring a Glc residue to GlcNAc modified Fap1 (17). The N-terminal domain of dGT1, which is referred as DUF1792 (domain of unknown function 1792), mediates the third step of Fap1 glycosylation by adding an additional Glc to Glc-GlcNAc modified Fap1 (15, 23). The other domain, the C terminus of dGT1 (C-dGT1), is responsible for the transfer of a second GlcNAc to Glc-Glc-GlcNAc modified Fap1, forming a branched tetrasaccharide, Glc-(GlcNAc)-Glc-GlcNAc (67). Like the C terminus of dGT1, both GalT2 and Gly were predicted to possess typical GT-A type glycosyltransferase domains. Our previous study also indicated that GalT2 is involved in the Fap1 glycosylation (22). However, their exact roles are yet to be determined. Fully glycosylated Fap1 is secreted with the assistance of several accessory secretion components including glycosylation-associated proteins Gap1, Gap2, and Gap3, as well as the accessory Sec components SecY2 and SecA2 (24–30).

In the present study, we engineered the Fap1 glycan biosynthetic pathway by co-expressing recombinant Fap1 with the well characterized and putative glycosyltransferases in *Escherichia coli*. We determined that GalT2 and Gly are the two remaining glycosyltransferases in the pathway that mediate the transfer of rhamnosyl and glucosyl residues, respectively. Furthermore, inactivation of each glycosyltransferase gene led to differentially impaired biofilms of *S. parasanguinis*, indicating the importance of the Fap1 glycosylation in the biofilm formation.

## Results

### 

#### 

##### GalT2 and Gly Catalyze the Sequential Glycosylation of Fap1 Following dGT1

**FIGURE 1.**
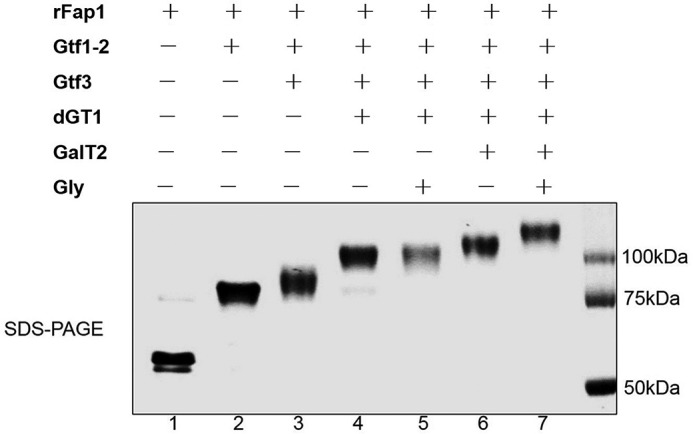
**Monitoring Fap1 modification in an *E. coli* glycosylation system.** Strains rFap1 (*lane 1*), rFap1-Gtf12 (*lane 2*), rFap1-Gtf123 (*lane 3*), rFap1-Gtf123-dGT1 (*lane 4*), rFap1-Gtf123-dGT1-Gly (*lane 5*), rFap1-Gtf123-dGT1-GalT2 (*lane 6*), and rFap1-Gtf123-dGT1-GalT2-Gly (*lane 7*) were induced, and differentially modified Fap1 variants were expressed and purified, respectively, and subjected to SDS-PAGE analysis.

To determine whether GalT2 and Gly can further modify Fap1, we engineered an *in vivo* glycosylation system in *E. coli* by co-expressing a recombinant Fap1 (rFap1) with a series of glycosyltransferases and then examined the modification of rFap1 variants by monitoring their migration using SDS-PAGE analysis. Because it is known that Gtf12, Gtf3, and dGT1 (DUF1972 and C-dGT1) mediate the first four steps of Fap1 glycosylation, respectively (14), we co-expressed either GalT2 or Gly with Gtf123-dGT1 modified rFap1. Unmodified rFap1 and differentially modified rFap1 proteins were purified and subjected to SDS-PAGE analysis and Coomassie Blue staining. As previously reported, a stepwise migration of rFap1 was observed that was caused by the sequential modification of rFap1 by Gtf12, Gtf3, and dGT1 (Fig. 1, *lanes 1–4*). In addition, GalT2 retarded the migration of Gtf123-dGT1 modified rFap1 (Fig. 1, *lane 6*), indicating the further modification of rFap1 by GalT2. Moreover, the migration of the modified rFap1 was further retarded when co-expressed with both GalT2 and Gly (Fig. 1, *lane 7*). However, such retardation was abolished when GalT2 was absent (Fig. 1, *lane 5*), suggesting that the further modification by Gly requires the presence of GalT2. These data indicate that dGT1 modified Fap1 was sequentially glycosylated by GalT2 and Gly, which constitutes the last two steps of the Fap1 glycosylation. The stepwise migration profile of these rFap1 variants (Fig. 1, *lanes 1–4*, *6*, and *7*) indicates the successful sequential modification of rFap1.

##### GalT2 and Gly Transfer Rha and Glc, Respectively, to Gtf123-dGT1 Modified rFap1

The mature Fap1 glycan has the sequence of Rha1–3Glc1-(Glc1–3GlcNAc1)-2,6Glc1–6GlcNAc (15). As demonstrated, Gtf12, Gtf3, and dGT1 are responsible for catalyzing the transfer of GlcNAc, Glc, Glc, and GlcNAc to Fap1, respectively (15, 17, 21). To determine how GalT2 and Gly contribute to the Fap1 glycosylation, both Gtf123-dGT1-GalT2 and Gtf123-dGT1-GalT2-Gly modified rFap1 were purified and subjected to glycan profiling, respectively. In this experiment, the glycans were reductively eliminated from the recombinant proteins, permethylated, and analyzed by MALDI-TOF (15).

**FIGURE 2.**
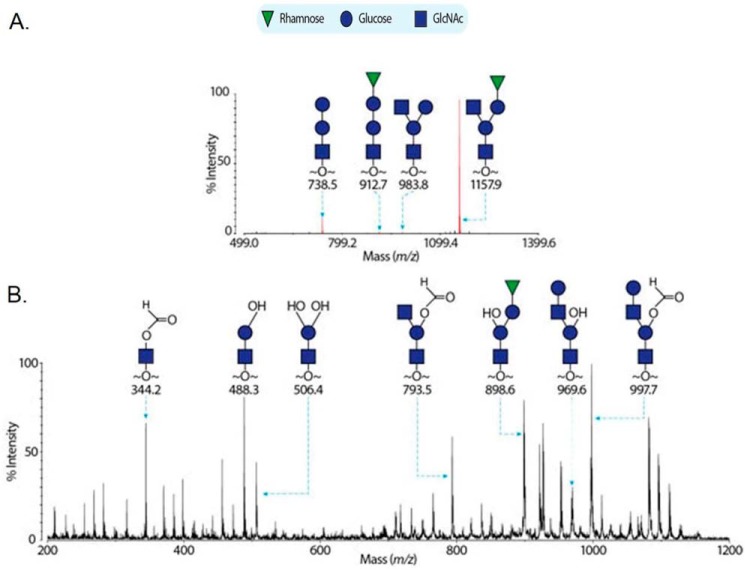
**Mass spectrometric analysis of rFap1 glycans in the presence of GalT2.** The MALDI-TOF spectrum of permethylated recombinant Fap1 glycans modified by Gtf1/2/3-dGT1-GalT2 is shown in *A*. The *red peaks* corresponding to glycans are annotated with *m*/*z* and structures. The MS/MS spectrum of the MS peak at *m*/*z* 1157.9 is shown in *B*.

When rFap1 is co-expressed with Gtf123-dGT1-GalT2, four MS peaks at *m*/*z* 738.8, 938.8, 912.7, and 1157.9 corresponding to glycans of compositions Glc-Glc-GlcNAc, Glc-(GlcNAc)-Glc-GlcNAc, Rha-Glc-Glc-GlcNAc, and Rha-Glc-(GlcNAc)-Glc-GlcNAc were detected, respectively (Fig. 2*A*). The former two peaks were previously observed when rFap1 was modified with Gtf123-dGT1. They correspond to a linear trisaccharide and a branched tetrasaccharide, respectively (15). The mass increments of 174 Da (from *m*/*z* 738.5 to 912.7 and from *m*/*z* 938.8 to 1157.9) indicated that the latter two peaks correspond to glycans formed by adding a deoxy-hexose to the former two glycans. Glycosyl composition analysis has revealed that the fully modified Fap1 glycan contains Rha as the sole deoxyhexose (15); thus we propose that GalT2 is a rhamnosyltransferase that adds a Rha to a Glc. In addition, GalT2 seems to have a semirelaxed specificity and can transfer a Rha to both linear and branched precursors, although it appears to favor the latter structure because of the observation that the peak at *m*/*z* 1157.9 dominated in Fig. 2*A*. To confirm the proposed annotations in Fig. 2*A*, the MS peak at *m*/*z* 1157.9 was further subjected to MALDI-TOF/TOF (Fig. 2*B*). The peak at *m*/*z* 793.5 and 898.6 strongly indicated that the peak at *m*/*z* 1157.9 corresponds to a branched pentasaccharide with GlcNAc and Rha-Glc antennae.

By comparing Fig. 2*A* with the published MS profiling of the mature Fap1 glycan (Gtf123-dGT1-GalT2-Gly modified rFap1) (15), we noticed that the peak at *m*/*z* 1157.9 shifts 204 Da to *m*/*z* 1366.2 when Gly is present. This mass increment is due to the addition of a Glc to the non-reducing end GlcNAc. Thus we propose Gly is a glucosyltransferase mediating the final step of Fap1 glycosylation.

**FIGURE 3.**
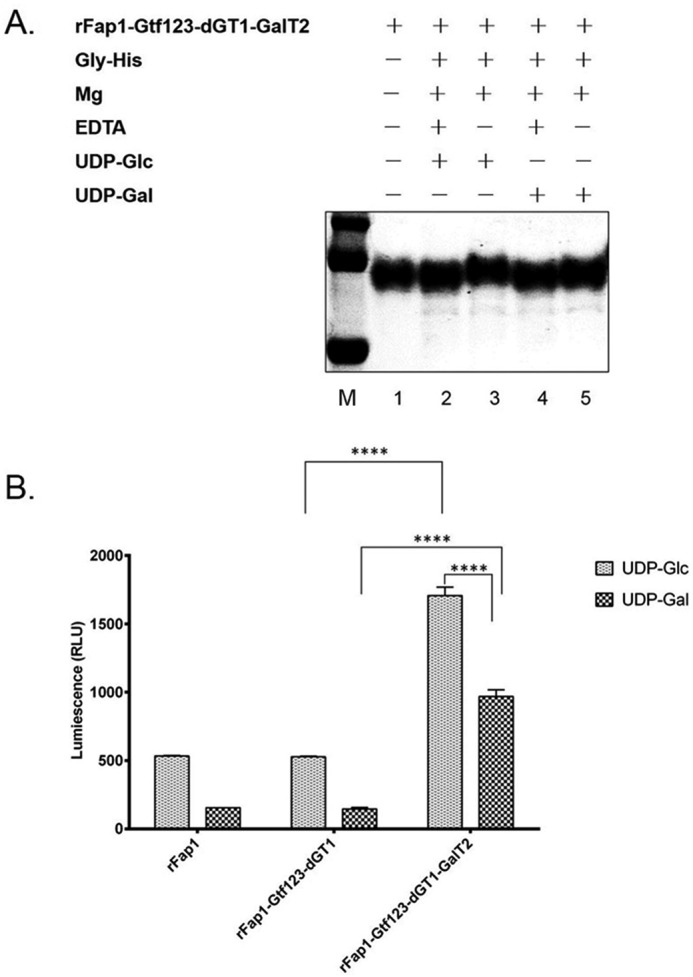
***In vitro* glycosyltransferase activity of Gly.** *A*, *in vitro* glycosylation gel shift assay. Purified enzyme His-Gly and the substrate Gtf123-dGT1-GalT2 modified rFap1 were added into 50 μl of reaction buffer (20 mm Tris, pH 8.0, 100 mm NaCl, and 10 mm Mg^2+^). 10 mm EDTA was used to chelate metal ions. 10 mm UDP-Glc or UDP-Gal was used as a sugar donor. The reaction mixture was incubated in test tubes in a 37 °C water bath for 15 h. Completed reactions were then processed for SDS-PAGE and Coomassie blue staining. *B*, UDP-Glo glycosyltransferase assay. 5 μl of glycosyltransferase reaction mixtures that contain recombinant Gly enzyme, rFap1, or rFap1-Gtf123-dGT1, or rFap1-Gtf123-dGT1-GalT2 as a substrate and UDP-Glc or UDP-Galactose as a sugar donor were incubated in a solid white 384-well plate. After incubating with UDP detection reagent, luminescence was recorded using a BioTek microplate reader. The values represent the means of three replicates. *RLU*, relative light units. The bar graphs represent the means ± S.D. values. ****, *p* < 0.0001; two-tailed unpaired Student's *t* test.

We also employed *in vitro* glycosyltransferase assays to confirm that Gly can directly transfer Glc to Gtf123-dGT1-GalT2 modified rFap1. We first incubated Gtf123-dGT1-GalT2 modified rFap1 with or without Gly, in the presence of UDP-Glc or UDP-Gal and then monitored the size shift of the samples by SDS-PAGE. As shown in Fig. 3*A*, there was a clear size shift when incubating with Gly, Mg^2+^, and UDP-Glc (*lanes 1–3*), suggesting that Gly can transfer UDP-Glc to Gtf123-dGT1-GalT2 modified rFap1. It is not surprising that the enzyme activity of Gly is metal ion-dependent because Gly has the typical GT-A type structural fold, and GT-A type glycosyltransferase needs metal ion to be active. Gly only has little affinity to Gal because there was no apparent shift observed when incubating with Gly, Mg^2+^, and UDP-Gal (*lanes 4* and *5*).

The glycosyltransferase activity of Gly was also determined by UDP-Glo glycosyltransferase assay through measuring the release of UDP caused by glycosylation reactions (31). Gly preferably transfers Glc to Gtf123-dGT1-GalT2 modified rFap1 but not to unmodified rFap1 or Gtf123-dGT1 modified rFap1 (Fig. 3*B*). Interestingly, compared with the other two acceptor substrates, Gly also seems to have an affinity toward Gtf123-dGT1-GalT2 modified rFap1 in terms of using UDP-Gal as a sugar donor, but the activity is lower (Fig. 3*B*). The affinity of Gly toward to UDP-Gal was observed in UDP-Glo assay (Fig. 3*B*) but not so apparent in the size shift assay (Fig. 3*A*), which could be due to the higher sensitivity of UDP-Glo assay. Nevertheless, the results from both *in vitro* assays are consistent with each other. It is worth noting that Gal was not detected from the carbohydrate composition analysis of the native Fap1 (15). Gly clearly has a preference in transferring Glc to Gtf123-dGT1-GalT2 modified Fap1 *in vivo*.

##### Gly Is Required for Fap1 Glycosylation and Maturation

**FIGURE 4.**
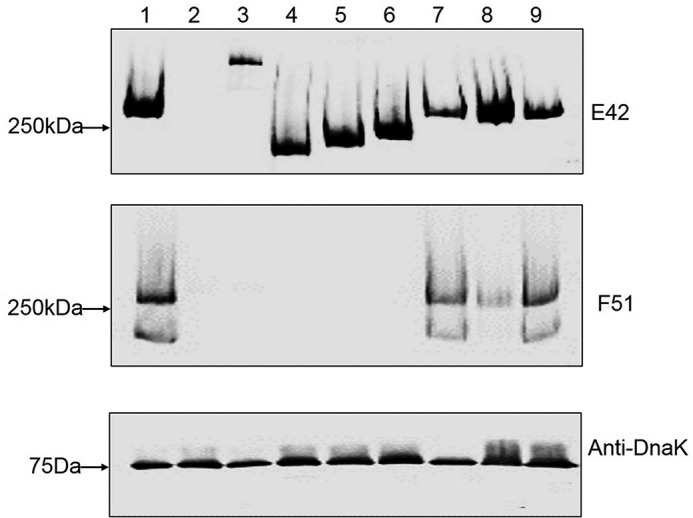
***gly* mutant exhibits Fap1 glycosylation defect.** Whole cell extracts prepared from the same number of bacterial cells of wild type (*lane 1*), *fap1* (*lane 2*), *gtf1* (*lane 3*), *gtf3* (*lane 4*), dG*T1* (*lane 5*), *galT2* mutant (*lane 6*), *galT2* complemented strain (*lane 7*), *gly* mutant (*lane 8*), and *gly* complemented strain (*lane 9*) were subjected to Western blotting analysis using Fap1 specific mAbs E42 (*top panel*) and F51 (*middle panel*), and anti-DnaK (*bottom panel*) as a sample loading control.

To further determine the relative contribution of Gly to the Fap1 glycosylation in the native host *S. parasanguinis*, a *gly* deletion mutant and its complemented strain were constructed and then examined for Fap1 production by Fap1 specific antibody mAb E42 and F51. Consistent with previous studies (17, 22), inactivation of *gtf1*, *gtf3*, *dGT1*, or *galT2* resulted in the production of Fap1 precursors that bind to the peptide specific mAb E42 with different migration patterns but failed to bind to mature Fap1 specific antibody F51 (Fig. 4, *top* and *middle panels*, *lanes 3–6*), suggesting that these Fap1 precursors possess glycosylation defects. The *gly* mutant produced one band binding to mAb E42 that migrated slightly faster than the band from either wild-type *S. parasanguinis* or the complemented *gly* strain (Fig. 4, t*op panel*, *lane 8 versus lanes 1* and *9*), indicating that this band is devoid of modification to some degree. Further, this band did not bind to the mature Fap1 specific antibody F51 as strong as that from either wild type *S. parasanguinis* or the complemented strain (Fig. 4, *middle panel*, *lane 8 versus lanes 1* and *9*), again suggesting the presence of the glycosylation defect. Interestingly, mature Fap1 migrated as a doublet, and both bands were recognized by the antibody F51 (Fig. 4, *middle panel*, *lanes 1*, *7*, and *9*). However, only the lower band is missing from the *gly* mutant (Fig. 4, *middle panel*, *lane 8*). Because the doublet was only observed in mature Fap1 (Fig. 4, *middle panel*, *lanes 1*, *7*, and *9*), we speculated that the doublet was associated the maturation form of Fap1. Inactivation of *gly* resulted in the disappearance of the lower band, indicating a subtle defect. The lower band could be a processed product from fully glycosylated Fap1 (*upper band*) but awaits further investigation. Nevertheless, these results suggest that Gly is required for the full maturation of Fap1.

##### Glycosyltransferases Differentially Affect Bacterial Biofilm Formation

**FIGURE 5.**
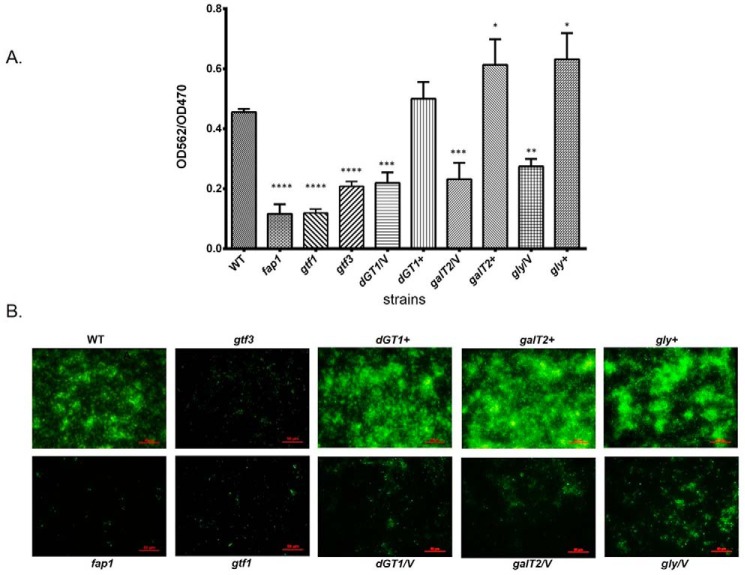
**Disruption of glycosyltransferases altered biofilm formation.** *A*, quantitative analysis of biofilms by microtiter plate. *In vitro* biofilm assays were performed as described under “Experimental Procedures.” Biofilms stained by crystal violet in the microtiter wells were extracted and quantified by measuring absorbance at 562 nm. The biofilms were normalized by bacterial growth using *A*_562_/*A*_470_. The *bars* represent the mean measurements from three independent experiments. *, *p* < 0.05; **, *p* < 0.01; ***, *p* < 0.001; ****, *p* < 0.0001 (two-tailed unpaired Student's *t* test). *B*, florescent microscopy analyses of biofilms. Biofilms of wild type (WT), fap1 mutant (*fap1*), gtf1 mutant (*gtf1*), gtf3 mutant (*gtf3*), dGT1 mutant (*dGT1*/V), *galT2* mutant (*galT2*/V), *gly* mutant (*gly*/V), and their complementary strains (*dGT1*+, *galT2*+, *gly*+) were formed on the μ-Slide 8-well plates, then stained with SYTO 9, and examined by florescent microscopy. *Bar*, 50 μm. /*V*, with an empty vector pVPT. +, complementation of a corresponding mutant.

To further investigate the role of dGT1 and Gly in *S. parasanguinis*, we examined the effect of either dG*T1* or *gly* mutation on the biofilm formation. The *galT2* mutant displayed a decreased biofilm biomass when compared with that of its parent strain or its complemented strain (Fig. 5*A*), which is consistent with previous findings (22). The d*GT1* mutant displayed a similar decrease in the biofilm formation as the *galT2* mutant (Fig. 5*A*). The *gly* mutant exhibited a minor decrease compared with that of other mutants (Fig. 5*A*), which might be due to the subtle defect of Fap1 glycosylation shown in Fig. 4. All the complemented strains restored the wild-type biofilm phenotype (Fig. 5*A*). It should be noted that all mutant strains have no defect in bacterial growth (data not shown). These results were further confirmed by fluorescence microscopy studies. Wild-type *S. parasanguinis* and all the complemented strains formed robust biofilms after 16 h (Fig. 5*B*). The dG*T1* mutant exhibited a pattern of reduced biofilms similar to the *galT2* mutant, and the *gly* mutant showed a slight decrease compared with that of other mutants (Fig. 5*B*). However, the pixel fluorescent intensity of those biofilms was reduced in comparison with that of the wild-type or complemented strains (Fig. 5*B*). In contrast, the *fap1* and *gtf3* mutants displayed a minimal attachment (Fig. 5*B*). Taken together, these results suggest that these glycosyltransferases play important roles in biofilm formation.

## Discussion

Glycosylation has emerged as an important bacterial post-translational modification 32). There are four glycosylation pathways that have been identified so far: oligosaccharyltransferase (OTase)-mediated N-glycosylation or *O-*glycosylation and stepwise *N*-glycosylation or *O-*glycosylation. In the OTase-mediated glycosylation pathways, the glycans are assembled first and then transferred “en bloc” from the lipid carriers onto acceptor proteins (2). However, in stepwise glycosylation pathways, the modification begins with the attachment of the first monosaccharide to an acceptor protein, and more sugar residues are then added one at a time to form mature glycans (2). OTase-mediated *N*-glycosylation from *Campylobacter jejuni* has been successfully engineered in *E. coli* (33). Since then, numerous efforts have made to develop bioconjugate vaccines *in E. coli* against pathogens such as *Francisella tularensis* (34), *Burkholderia pseudomallei* (35), *Shigella flexneri* (36), and *S. aureus* (37) using this system. An OTase-mediated *O-*glycosylation system from *Neisseria meningitides* was established in *Shigella* spp. to produce conjugate vaccines (38). OTase-mediated *O-*glycosylation systems from *Pseudomonas aeruginosa* 1244 and *N. meningitidis* MC58 have been expressed in *E. coli* (39). However, in both cases, sugars are transferred en bloc by OTase named PglL in *N. meningitidis* and PilO in *P. aeruginosa.* There is no reported case of the re-establishment of the exogenous stepwise *O-*glycosylation pathway in *E. coli*.

**FIGURE 6.**
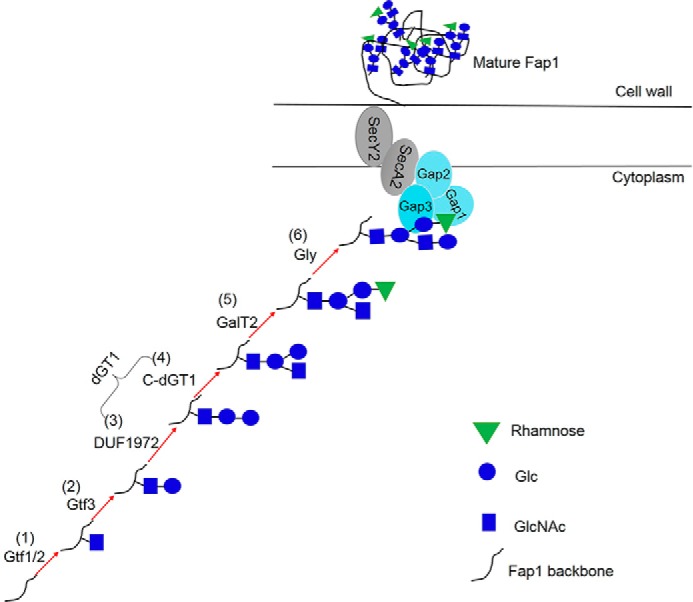
**A model for the Fap1 biogenesis in *S. parasanguinis*.** Fap1 is sequentially glycosylated by Gtf12 (*1*), Gtf3 (*2*), DUF1972 (*3*), C-dGT1 (*4*), GalT2 (*5*), and Gly (*6*) to produce fully modified Fap1. The fully modified Fap1 is then processed, secreted, and anchored to cell wall with the assistance of an accessory secretion protein complex (Gap1, Gap2, and Gap3) and secretion components SecA2 and SecY2.

In the current study, we demonstrated the successful engineering of stepwise *O-*linked protein glycosylation pathway in *E. coli*. Genetic, biochemical, and structural approaches have allowed us to characterize the sequential Fap1 glycosylation pathway (Fig. 6) by reconstituting the machinery in *E. coli*. The glycan profile of fully modified Fap1 from *E. coli* corresponds well with that of the mature Fap1 purified from *S. parasanguinis* (15), indicating the successful glycoprotein engineering in our studies.

A similar biosynthetic gene cluster mediates the glycosylation of various SRRPs (40). The initial step of SRRP glycosylation mediated by a two-protein glycosylation complex Gtf12 (or GtfAB) is highly conserved in streptococci, staphylococci, and lactobacilli, indicating *O-*GlcNAc is a core glycan structure of SRRPs (18, 21, 41, 42). The second step of SRRP glycosylation mediated by Gtf3 (or GtfC) is semiconserved that has only been reported in streptococci. It is worth noting that the organization and number of putative glycosyltransferases found in the *srrp* gene clusters are quite diverse (6). For instance, *S. pneumoniae* possesses many more annotated glycosyltransferases than *S. aureus* (6). Such diversity of glycosyltransferases may enable the bacteria to modify adhesion molecules for their unique host environment. In addition, the current report of the reconstitution of the Fap1 glycosylation pathway in *E. coli* offers an excellent model to elucidate the glycosylation pathways of other SRRPs, which would also help expand the glycoengineering toolbox to facilitate the production of recombinant glycoproteins with industrial and medical purposes (38, 43–47).

Along with the growing investigation of bacterial protein glycosylation, there have been increased numbers of reports of glycosyltransferases in the past few decades (3, 14, 43, 48). The classification of glycosyltransferases was normally based on the similarities of amino acid sequences (49). However, the alignment is often limited to the conserved catalytic domain, which does not warranty the adequate identification of specific activity conferred by each glycosyltransferase. For instance, although GalT2 shares 40% amino acid sequence identity with dGT1 (both GalT2 and C-dGT1 have the classic GT-A type structural fold), the function of each enzyme is distinctively different. dGT1 is a glycosyltransferase with dual functions that transfer both Glc and GlcNAc, whereas GalT2 is responsible for the transfer of rhamnose. Because of the distinct activity of dGT1 and GalT2 and their high sequence similarity, it will be interesting to compare the structural details of the two enzymes to understand the mechanism underlying the sugar specificity. Structural characterization of GalT2 is currently ongoing.

GalT2 is the first rhamnosyltransferase reported to be involved in the glycosylation of a bacterial adhesin, because the other few known rhamnosyltransferases are either involved in cell wall biogenesis in plants or in the synthesis of lipopolysaccharides or capsule polysaccharides in bacteria (50–54). In bacteria, rhamnose is often one of the main components of cell surface polysaccharides and is synthesized from dTDP-rhamnose (55). Much effort has been undertaken to study the gene cluster responsible for the biosynthesis of the rhamnose donor, dTDP-Rha from dTDP-glucose (dTDP-Glc) (53). It is worth noting that such a gene cluster also exists in *S. parasanguinis*, indicating the availability of dTDP-Rha *in vivo*. Unfortunately, we could not perform an *in vitro* biochemical assay because of the lack of the sugar donor, dTDP-Rha. However, GalT2 did not catalyze the transfer of Glc, GlcNAc, GalNAc, or Gal when their respective UDP sugars were used in established *in vitro* glycosyltransferase assays (data not shown). Nevertheless, our mass spectrometry data strongly suggest that GalT2 is a rhamnosyltransferase in the glycosylation of Fap1. Therefore, we will rename GalT2 as Rha1 for the future use.

Gly also has the typical GT-A type structural fold and shares up to 50% sequence identity to GalT2. Both mass spectrometry analysis and an *in vitro* glycosyltransferase assay reveal that Gly is not a rhamnosyltransferase (Figs. 2 and 3). Interestingly, Gly seems to have a relaxed specificity with respect to sugar donors because it can transfer both Glc and Gal in the *in vitro* glycosyltransferase assay. Because no galactose, only additional glucose, was found in the fully glycosylated Fap1 purified from *E. coli* or *S. parasanguinis* (15), and Gly indeed has higher activity toward transferring glucose, we conclude that Gly functions as a glucosyltransferase in the glycosylation of Fap1.

Biofilm formation can be divided into three distinct steps: initial attachment of bacteria to a surface, microcolony formation, and biofilm maturation. Fap1, as a surface adhesin, is required for biofilm formation (4), and the glycosylation of Fap1 plays an important role. Inactivation of the *fap1* gene completely abolished bacterial attachment to saliva-coated hydroxylapatite surfaces and biofilm formation (5). The *gtf1* defect exerted the same impact on the biofilm formation as the *fap1* deletion did (Fig. 5), which is likely due to the fact that the *gtf1* mutant could not produce stable Fap1 protein (22). Inactivation of *gtf3*, *dGT1*, *galT2*, or *gly* reduced bacterial ability to form biofilms (Fig. 5) to different degrees. Cell wall fraction analysis of various *gtf* mutants in *S. parasanguinis* indicated that only the removal of the initial sugar (*gtf1* and *gtf2* mutant) resulted in little or no Fap1 transport to cell wall (24). A comparable amount of differentially modified Fap1 from other *gtf* mutants was found transported to the cell wall, suggesting that the biofilm defects are likely due to the glycosylation defects seen in those *gtf* mutants. Extracellular matrix exopolysaccharides affect bacterial biofilm development and biomass accumulation in bacteria (22). It is possible that glycosylated Fap1 functions as a substitute of exopolysaccharides to glue bacteria layers together. The *gtf3*, *dGT1*, *galT2*, or *gly* deletion mutant may have produced a less optimal intercellular matrix for microcolony formation than wild-type bacteria.

Biofilm formation plays a prominent role in bacterial pathogenesis (40, 56). The crucial role played by protein glycosylation in the biofilm formation is common in many bacterial pathogens. For instance, protein *O-*glycosylation is important in the phytopathogen *Ralstonia solanacearum* as the disruption of the *O-*oligosaccharyltransferase led to a defect in the biofilm formation and reduced pathogenicity (57). An *O-*glycosylation system identified in a human pathogen *Acinetobacter baumannii* was also shown to be important in biofilm formation and bacterial virulence (58). However, these studies have not systematically dissected each individual glycosyltransferase and how they are involved in these glycosylation systems. Interestingly, inactivation of some glycosyltransferase genes in gut bacteria has also been shown to affect the biofilm formation (59, 60). Deletion of glycosyltransferase bgsB of *Enterococcus faecalis* leads to a complete loss of glycolipids from the cell membrane, as well as impaired biofilm formation and reduced virulence *in vivo* (59). Inactivation of glucosyltransferase A (GtfA) from *Lactobacillus reuteri* TMW1.106 results in decreased biofilm formation and bacterial colonization in the murine gut, although the targeted substrates of GtfA are unknown (60). Nevertheless, both glycosyltransferases and protein glycosylation play important roles in biofilm formation and bacterial pathogenesis.

In summary, the current study reported the first complete *O-*glycosylation pathway of SRRPs, which is the first reconstitution of a stepwise *O-*linked protein glycosylation system in *E. coli*. We further defined the role of each glycosyltransferase in the Fap1 glycosylation (Fig. 6) and biofilm formation. Because Fap1-like SRRPs and the genes responsible for SRRP glycosylation are conserved in streptococci and lactobacilli, and the modification of these glycoproteins are critical for the bacterial-host interactions (3, 6, 14, 40, 61, 62), the defined glycosylation pathway presented in this report can aid in studying the glycosylation mechanisms of other SRRPs. This will shed light on the development of new therapeutic agents for the prevention and treatment of bacterial infections.

## Experimental Procedures

### 

#### 

##### Bacterial Strains, Plasmids, and DNA Manipulation

Bacterial strains and plasmids used in this study are listed in Table 1. Antibiotics were used at the following concentrations: 10 μg/ml erythromycin and 150 μg/ml kanamycin in Todd-Hewitt (TH) broth or agar plates for *S. parasanguinis*; 300 μg/ml erythromycin, 50 μg/ml kanamycin, and 250 μg/ml chloramphenicol in LB broth; or agar plates for *E. coli*. Isolation of plasmid DNAs was carried out with a QIAprep miniprep kit (Qiagen). PCR was carried out with *Taq* DNA polymerase (Promega) or KOD DNA polymerase (Novagen). PCR products were purified with a QIAquick PCR purification kit (Qiagen). DNA digestion, ligation, and transformation were performed using standard methods.

**TABLE 1:** **Strains and plasmids used in the study**

	Relevant properties	Source/reference
**Strains**		
*S. parasanguinis* FW213	Wild type	
*E. coli* Top10	Host for propagation of recombinant plasmids	Invitrogen
*E. coli* BL21 Gold (DE3)	pET system host strain	Invitrogen
*fap1* mutant	Wild type; *fap1*::*aphA3*; Kan^r^	Ref. 5
*gtf1* mutant	VT508	Ref. 64
*gtf3* mutant	Wild type; *gtf3* knockout; *gtf3*::*aphA3*; Kan^r^	Ref. 17
*dGT1* mutant	Wild type; d*GT1* knockout; dG*T1*::*aphA3*; Kan^r^	Ref. 15
*galT2* mutant	Wild type; *galT2* knockout; *galT2*::*aphA3*; Kan^r^	Ref. 22
*gly* mutant	Wild type; *gly* knockout; *gly*::*aphA3*; Kan^r^	This study
His-Gly	pET28b-Gly in BL21	This study
rFap1	pET28a-rFap1-R1 in BL21	Ref. 15
rFap1-Gtf123-dGT1-Gly	pET28a-rFap1-R1 and pvpt-Gtf123-dGT1-Gly and pHSG576 co-transformed into BL21	This study
rFap1-Gtf123-dGT1-GalT2	pET28a-rFap1-R1 and pvpt-Gtf123-dGT1-GalT2 and pHSG576 co-transformed into BL21	This study
rFap1-Gtf123-dGT1-GalT2-Gly	pET28a-rFap1-R1 and pvpt-Gtf123-dGT1-GalT2 and pHSG576-Gly co-transformed into BL21	Ref. 15

**Plasmids**		
pET28a	His tag fusion protein expression vector Kan^r^	Invitrogen
pHSG576	PCR cloning vector; Cmr	Ref. 5
pALH124	aphA3 Kanr cassette-containing plasmid; Amp^r^ Kan^r^	Ref. 65
pVPT-CHSV	*E. coli-Streptococcus* shuttle vector; Erm^r^	Ref. 66
pVPT-Gtf123-dGT1-Gly	Gtf123, dGT1 and Gly cloned into pVPT-CHSV; Erm^r^	This study
pET28b-Gly	Gly cloned into pET28b	This study
pVPT-Gly	Gly cloned into pVPT-CHSV; Erm^r^	This study

##### Engineering the Fap1 Glycosylation Pathway in E. coli

To obtain differentially modified Fap1, plasmid pVPT-Gtf12, pVPT-Gtf123, pVPT-Gtf123-dT1, pVPT-Gtf123-dGT1-Gly, or pVPT-Gtf123-dGT1-GalT2 was co-transformed with pET-28a-rFap1-R1 and pHSG576, respectively, into BL21 to produce the following strains rFap1-Gtf12, rFap1-Gtf123, rFap1-Gtf123-dGT1, rFap1-Gtf123-dGT1-Gly, and rFap1-Gtf123-dGT1-GalT2. To produce fully modified Fap1, plasmid pVPT-Gtf123-dGT1-GalT2 was co-transformed with pET-28a-rFap1-R1 and pHSG576-Gly into BL21, resulting in strain rFap1-Gtf123-dGT1-GalT2-Gly. All modified Fap1 were expressed and purified using the same method described previously (15). The plasmids and strains constructed are listed in Table 1. All plasmids were confirmed by DNA sequencing.

##### Reductive Elimination and Permethylation

Purified modified rFap1-R1 variants were subjected to glycan profiling as described previously (15). In brief, each freeze-dried rFap1 sample and 22 mg of potassium borohydride were dissolved in a 400 μl of 0.1 m potassium hydroxide solution. The solution was incubated at 45 °C for 18 h, and 5–6 drops of acetic acid were added to quench the reaction. The solution was loaded onto a 50E-8C Dowex® column and eluted with 5% acetic acid. The collected eluent was concentrated and lyophilized. Excessive borates were co-evaporated with 10% methanolic acetic acid. For permethylation, sodium hydroxide (3–5 pellets/sample) was crushed in 3 ml of dry dimethyl sulfoxide. The resulting slurry and methyl iodide (each 750 μl) were added to the sample, and the mixture was agitated for 30 min. 2 ml of ultrapure water was then added to the mixture with shaking for quenching the reaction. The permethylated glycans were subsequently extracted with 2 ml of chloroform and washed twice more with ultrapure water. Chloroform was removed under a stream of nitrogen. The permethylated glycans were loaded onto a conditioned C18 Sep-pak® column, washed with 5 ml of ultrapure water, and successively eluted with 3 ml each of 15, 35, 50, and 75% aqueous acetonitrile. These solutions were collected and lyophilized.

##### MALDI-TOF and MALDI-TOF/TOF Analysis

MS spectra were recorded by using either a Voyager DE-STRTM MALDI-TOF or a 4800 plus MALDI-TOF/TOF mass spectrometer (Applied Biosystems, Darmstadt, Germany). MS/MS spectra were obtained by using the latter instrument. The MS mode was calibrated with a 4700 Calibration standard kit (Applied Biosystems), and the MS/MS mode was calibrated with fibrinopeptide B, human (Sigma). The collision energy for CID MS/MS was set to 1 kV, and the collision gas was argon. 2,5-Dihydroxybenzoic acid was used as matrix. Permethylated glycans were dissolved in 10 μl of methanol, and 1 μl of this solution was premixed with 1 μl of matrix and spotted onto the MALDI plate for further analysis.

##### In Vitro Glycosyltransferase Assay

To confirm the glycosyltransferase activity of Gly, two different *in vitro* assays were performed. Recombinant Gly enzyme was expressed and purified as described previously (15). For gel shift assay, 15 μl of purified His-Gly (20 μm) and 10 μl of Gtf123-dGT1-GalT2 modified rFap1 (50 μm) were added into 50 μl of reaction buffer (20 mm Tris, pH 8.0, 100 mm NaCl, and 10 mm Mg^2+^). 10 mm EDTA was used as metal ion chelator. 10 mm UDP-Glc or UDP-Gal was used as sugar donor. The mixture was incubated in test tube at 37 °C water bath for 15 h. Then sample loading buffer was added to the samples for SDS-PAGE.

For the UDP-Glo Glycosyltransferase Assay (Promega) (31), 5 μl of the glycosyltransferase reaction mixtures that contain 20 μm recombinant Gly enzyme, 50 μm rFap1, or rFap1-Gtf123-dGT1, or rFap1-Gtf123-dGT1-GalT2 as a substrate and 25 μm UDP-Glc, or UDP-Gal were incubated in a solid white 384-well plate. After a 1-h incubation at room temperature, 5 μl of UDP detection reagent was added to each well. After another 1-h incubation at room temperature, luminescence was recorded using a BioTek Microplate reader. The values represent the mean of three experimental replicates.

##### Construction of a Gly Knock-out Mutant and Its Complemented Strain

A non-polar *gly* knock-out mutant was generated by insertional mutagenesis with a kanamycin resistance cassette (Kan^r^). Briefly, the *gly* gene and its flanking regions including the 600-bp upstream and 600-bp downstream regions were amplified from genomic DNA of *S. parasanguinis* FW213. The PCR fragment was purified and cloned into pGEM-T Easy vector (Promega, Madison, WI). An 800-bp *gly* internal fragment was replaced with an 830-bp non-polar kanamycin resistance cassette (*aphA3*) isolated from pALH12462 by an inverse PCR strategy. Plasmid was confirmed by sequencing and then transformed into the FW213 strain by electroporation. Transformants were selected on TH agar plates containing kanamycin. The *gly* allelic replacement mutant was selected by its ability to resist kanamycin and further verified by PCR and sequencing analysis. The confirmed *gly* allelic replacement mutant was used in this study.

To complement the *gly* mutant, the full-length *gly* gene was amplified from FW213 genomic DNA by PCR using a primer set Gly-SalI and Gly-KpnI. The purified *gly* PCR product was digested with SalI and KpnI and then cloned into *E. coli-Streptococcus* shuttle vector pVPT-CHSV to generate the *gly* complementation plasmid pVPT-Gly. The plasmid and its control vector pVPT-CHSV were then transformed into the *gly* mutant via electroporation. The transformants were selected on TH agar plates containing kanamycin and erythromycin.

##### Western Blotting Analysis

For all *S. parasanguinis* strains, bacteria grown to an *A*_470_ value of 0.8–0.9 were harvested by centrifugation. The cell pellets were treated with amidase to lyse the cells (20). For *E. coli* strains, bacteria grown to an *A*_600_ value of 0.6–0.7 were harvested by centrifugation. Cell lysates were prepared by boiling the cell pallets collected in sample buffer (0.0625 m Tris HCl, pH 6.8, 2% SDS, 10% glycerol, 0.01% bromphenol blue) for 10 min and then analyzed using 6% or 8% SDS-PAGE and subjected to Western blotting. For *S. parasanguinis* samples, two monoclonal mAbs (63) were used to detect Fap1: mAb E42 (1:3000), the Fap1 peptide specific antibody, and mAb F51 (1:5000), the mature Fap1 specific antibody. Anti-DnaK was used as a sample loading control to detect a conserved chaperone DnaK in *S. parasanguinis*.

##### Biofilm Formation Assays

Biofilm formation of *S. parasanguinis* was first assessed using microtiter plate assays. Overnight cultures of *S. parasanguinis* were diluted to 1:100 in TYE broth with 1% sucrose, and 200 μl of each culture were inoculated into wells of sterile 96-well polystyrene microtiter plates (Nunc) and incubated at 37 °C in 5% CO_2_ for 16 h. The optical density of cells at 470 nm was used to monitor bacterial growth. The biofilm formation on each well was stained with 0.1% crystal violet and measured at 562 nm as previously described (22). Biofilm formation was also evaluated by fluorescence microscopy. The 1:100 diluted overnight cultures of *S. parasanguinis* strains were transferred into wells of sterile μ-Slide 8-well plates (ibidi). Biofilms formed on the slides over a period of 16 h were then gently rinsed three times with PBS and stained with SYTO 9 (Molecular Probes). The stained biofilms were imaged using Nikon ellipse 90i microscope equipped with an Epi-fluorescence and NIS elements AR. Biofilms of each strain were scanned from representative areas and recorded.

## Author Contributions

F. Z. and H. W. designed the study; F. Z. and H. Z. performed the *in vitro* and *in vivo* experiments; F. Z., H. Z., and H. W. analyzed the experimental data; T. Y. performed glycan structure analysis; T. Y., S. M. H., and A. D. analyzed the MS data; F. Z., H. W., A. D., S. M. H., and T. Y. wrote the paper. All authors approved the final version of the manuscript.

## Footnotes

3 The abbreviations used are:

SRRP: serine-rich repeat glycoprotein
OTase: oligosaccharyltransferase
TH: Todd-Hewitt.
